# Supplementary material for: AcrIF9 tethers non-sequence specific dsDNA to the CRISPR RNA-guided surveillance complex
Source: Nat Commun. 2020 Jun 1;11:2730. doi: 10.1038/s41467-020-16512-1 (PMC7264359; doi:10.1038/s41467-020-16512-1)
Supplement: Supplementary file 1 — Supplementary Information [file 41467_2020_16512_MOESM1_ESM.docx]

**Supplementary Information**

AcrIF9 Tethers Non-Sequence Specific dsDNA to the CRISPR RNA-guided Surveillance Complex

Marscha Hirschi, Wang-Ting Lu et al.

**Supplementary Fig. 1**
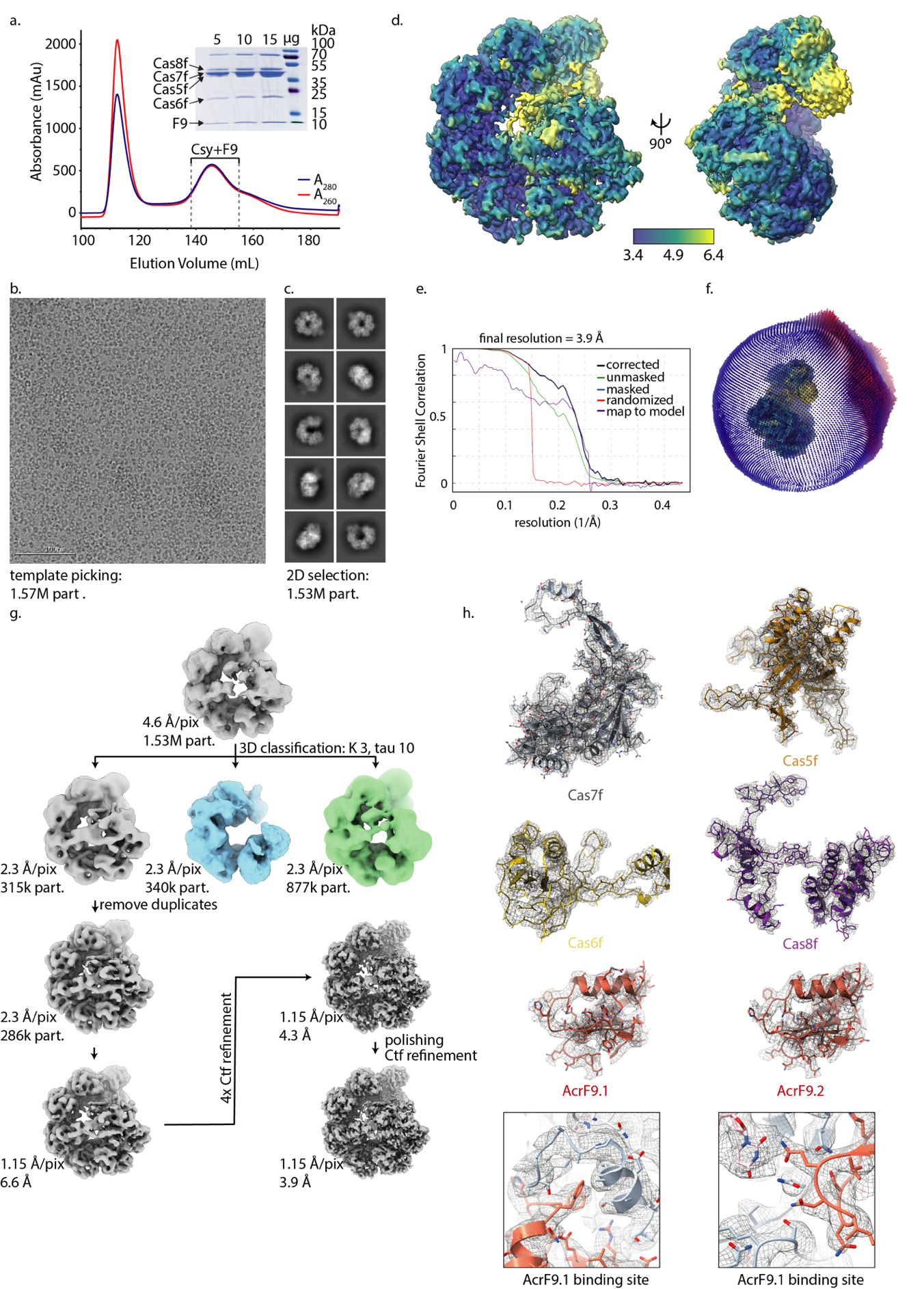
**Supplementary Fig. 1 Sample preparation, imaging, and processing of Csy-AcrIF9.** **a.** Size exclusion chromatography (Superdex 200 26/600, GE Healthcare) profile and SDS-PAGE analysis of the Csy-AcrIF9 complex. Source data are provided as a Source Data file. **b.** Representative micrograph of the specimen tilted to 30º. **c.** Selected 2D classes showing secondary structural information. **d.** Local resolution of the Csy-AcrIF9 reconstruction, calculated using Bsoft^15^. **e.** Curves calculated using the half maps, as well as the Fourier Shell Correlation between the atomic model and the final map (purple). **f.** Euler distribution for the final reconstruction. **g.** Data processing scheme for Csy-AcrIF9. After 2D clean-up the particle stack was refined at binning 4 x 4. The refined particles were re-centered and re-extracted at binning 2 x 2. One high-resolution class, containing 315k particles, was obtained by 3D classification (3 classes, tau-fudge 10). Duplicate particles were removed, leaving 286k particles that were re-centered and re-extracted unbinned. The unbinned refinement resulted in a reconstruction with a nominal resolution of ~6.6 Å. Four rounds of CTF refinement improved the nominal resolution to ~4.3 Å. Bayesian polishing followed by one final round of CTF refinement was performed to obtain a final reconstruction at a nominal resolution of 3.9 Å. **h.** EM density quality of Csy-AcrIF9. Components of the Csy-AcrIF9 complex fit into the EM density, EM density shown in mesh, molecular models shown in cartoon representation with side chains shown as sticks, crRNA shown in stick representation, colored as in Fig. 1.

**Supplementary Fig. 2**


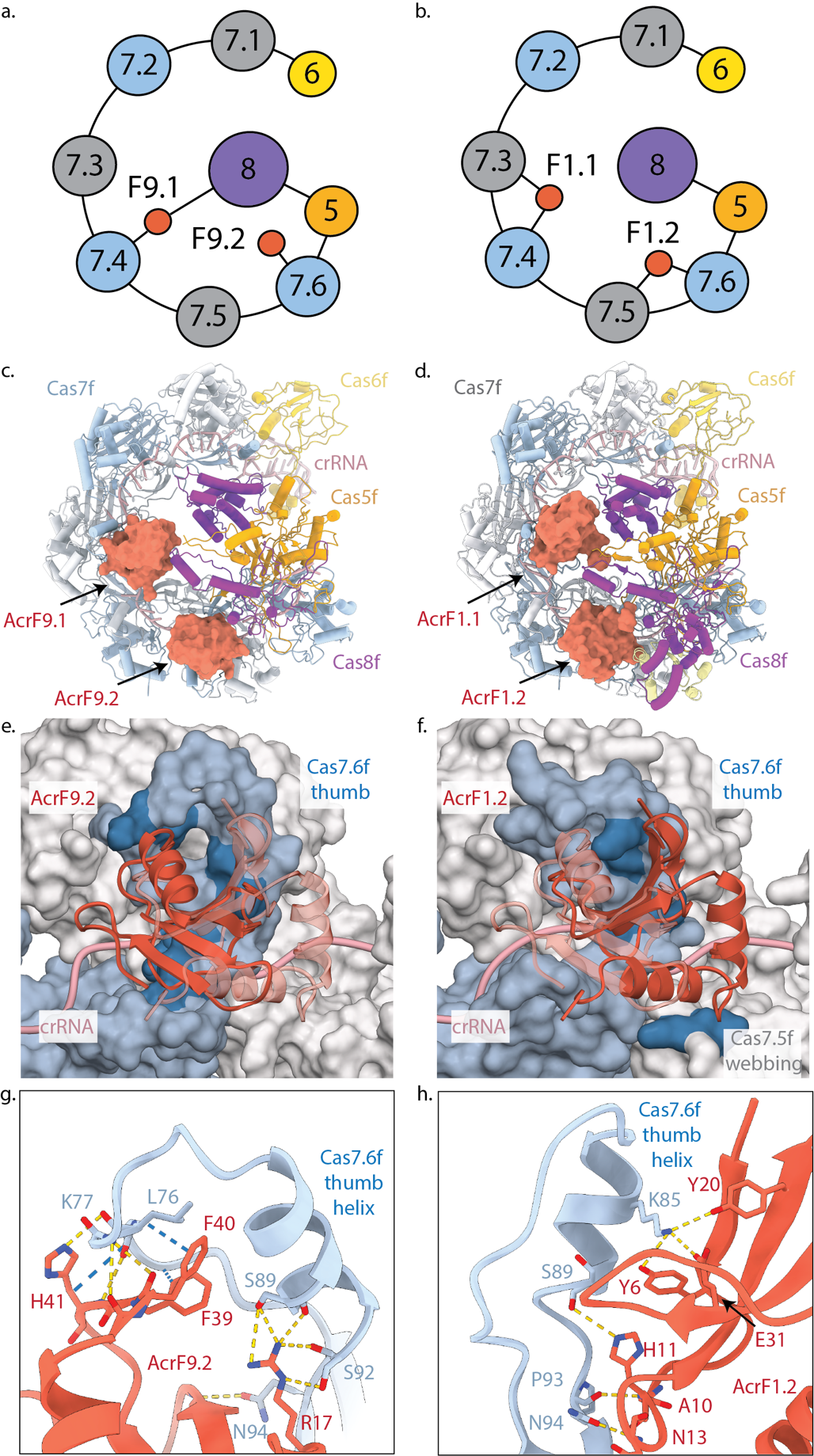


**Supplementary Fig. 2 Comparison of AcrIF9 and AcrIF1.** **a-b.** Schematic of the Csy-AcrIF9 (a) and Csy-AcrIF1 (b) complexes, with subunits colored as in Fig. 1. While AcrIF9 interacts with a single Cas7f, AcrIF1 interacts with residues from two neighboring Cas7f molecules. Inter-subunit interactions are denoted by lines. **c-d.** Model of the Csy-AcrIF9 complex (c) and Csy-AcrIF1/AcrIF2 complex (d), Csy subunits shown as pipes and planks, Acrs shown in surface representation. **e-f.** The binding site of AcrIF9.2 (e) and AcrIF9.1 (f), residues interacting with the Acr highlighted in dark blue. AcrIF9 is composed of a five-stranded anti-parallel beta sheet, cradling an alpha helix. AcrIF1 is composed of a four-stranded anti-parallel beta sheet, flanked on one side by two alpha helices. The folds of the Acrs are notably different. In order to illustrate the difference between Acr binding sites, AcrIF1.2 is shown transparent in e. and AcrIF9.2 is shown transparent in f. Cas7f subunits shown in surface representation, crRNA and Acrs in cartoon representation. **g-h.** Detailed view of the interaction interface in the region of the Cas7.6f thumb helix for AcrIF9 (g) and AcrIF1 (h). While the AcrIF9 and AcrIF1 binding sites overlap, the majority of interactions with Csy residues are different, only interactions with S89 and N94 are common to both Acrs. Model shown in cartoon representation, interacting residues shown as sticks, hydrogen bonds are indicated by yellow dashes, hydrophobic interactions by blue dashes.

**Supplementary Fig. 3**


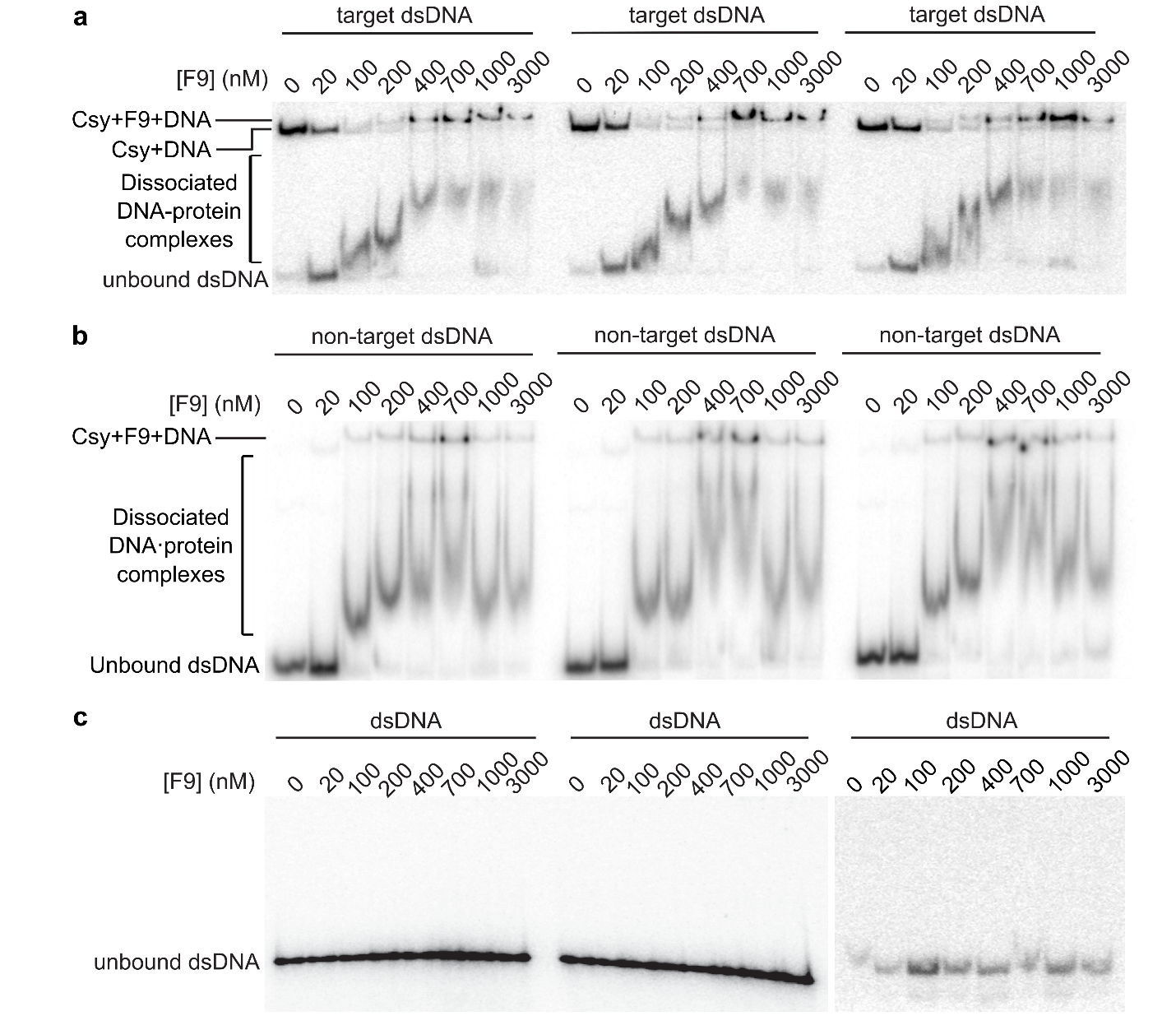


**Supplementary Fig. 3 Csy-AcrIF9 complex binds to dsDNA.** Electrophoretic mobility shift assays performed in triplicate. Source data are provided as a Source Data file. **a.** Csy-AcrIF9 binds target and (**b**) non-target DNA. Csy (200 nM) was incubated for 15 min with increasing concentrations of AcrIF9 prior to addition of ^32^P-labeled target or non-target DNA. **c.** AcrIF9 does not bind dsDNA alone.

**Supplementary Fig. 4**


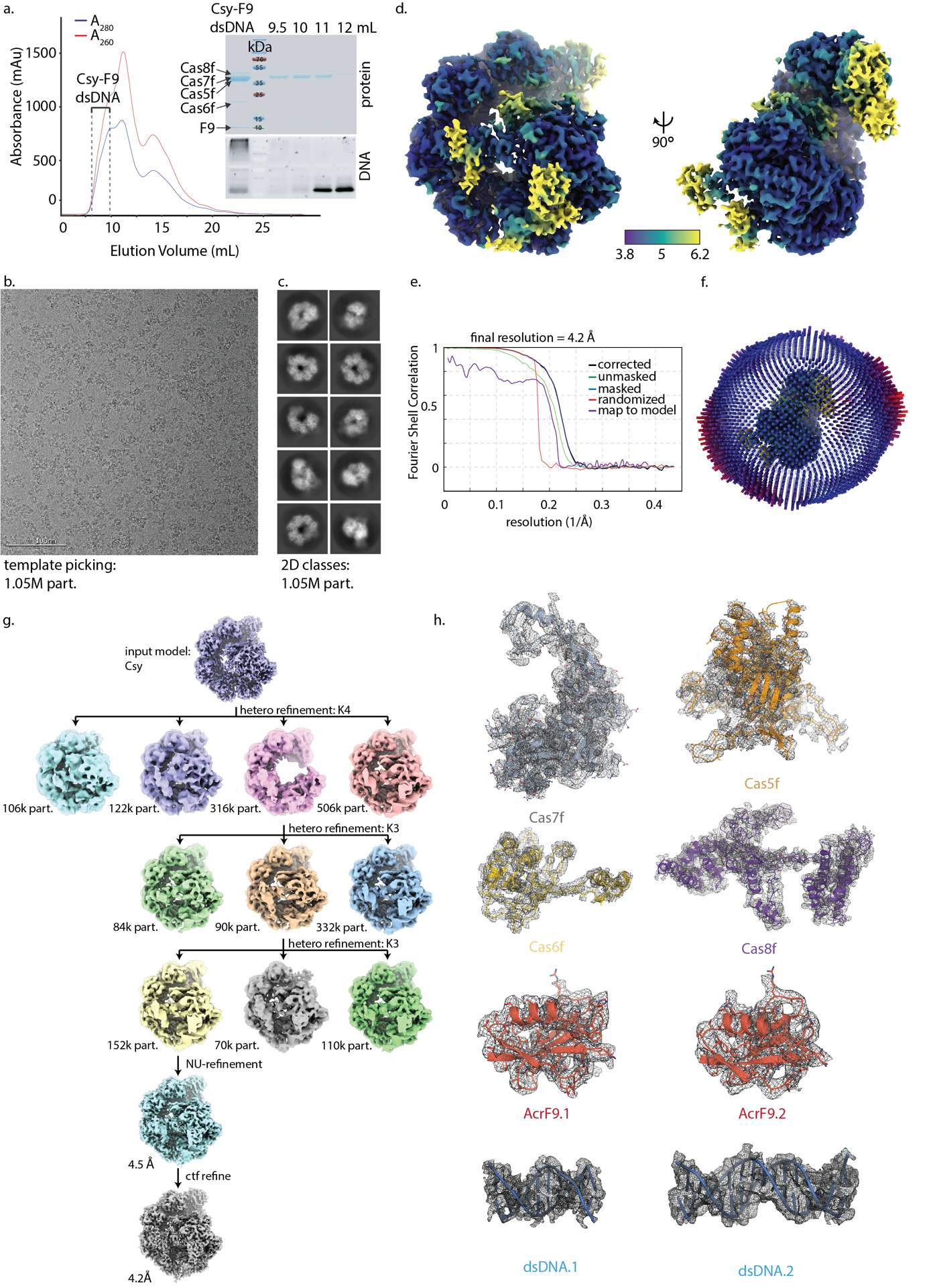
**Supplementary Fig. 4 Sample preparation, imaging, and processing of Csy-AcrIF9-dsDNA.** **a.** Size exclusion chromatography (Superdex 200 10/300, GE Healthcare) profile and SDS-PAGE analysis of the Csy-AcrIF9-dsDNA complex. Source data are provided as a Source Data file. **b.** Representative micrograph of the specimen. **c.** Selected 2D classes. **d.** Local resolution of the Csy-AcrIF9-dsDNA reconstruction calculated using Bsoft^15^. **e.** Fourier Shell Correlation curves calculated between the half maps. **f.** Euler distribution for the final reconstruction. **g.** Cryo-EM data processing scheme for Csy-AcrIF9-dsDNA. After particle pick inspection, the particles were heterogeneously refined into four classes, one class containing high resolution structural features was selected. An additional three rounds of heterogeneous refinement resulted in a particle stack of 106k particles that was refined using non-uniform refinement. This particle stack was imported into Relion and subjected to three rounds of CTF refinement. **h.** EM density quality of Csy-AcrIF9-dsDNA. Components of the Csy-AcrIF9-dsDNA complex fit into the EM density, EM density shown in mesh, molecular models shown in cartoon representation with side chains shown as sticks, crRNA shown in stick representation, colored as in Fig. 1.

**Supplementary Fig. 5**

**
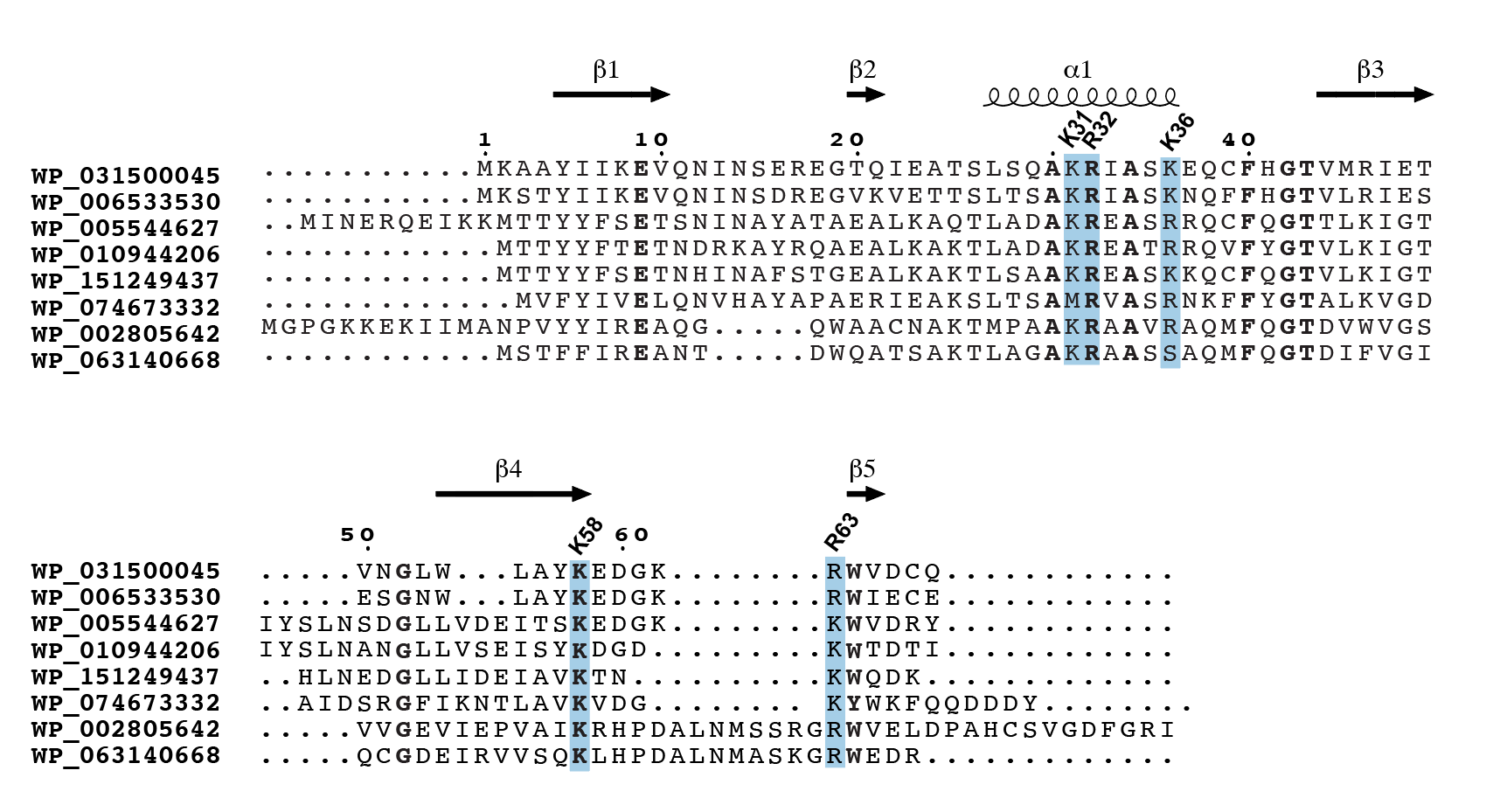
**

**Supplementary Fig. 5 Multiple sequence alignment of AcrIF9s.** AcrIF9 homologues were aligned using MAFFT^21^. Secondary structural elements are shown above the sequence. Conserved residues are shown in bold and positively charged residues close to the dsDNA are numbered above the sequence and highlighted in blue.

**Supplementary Fig. 6**

**
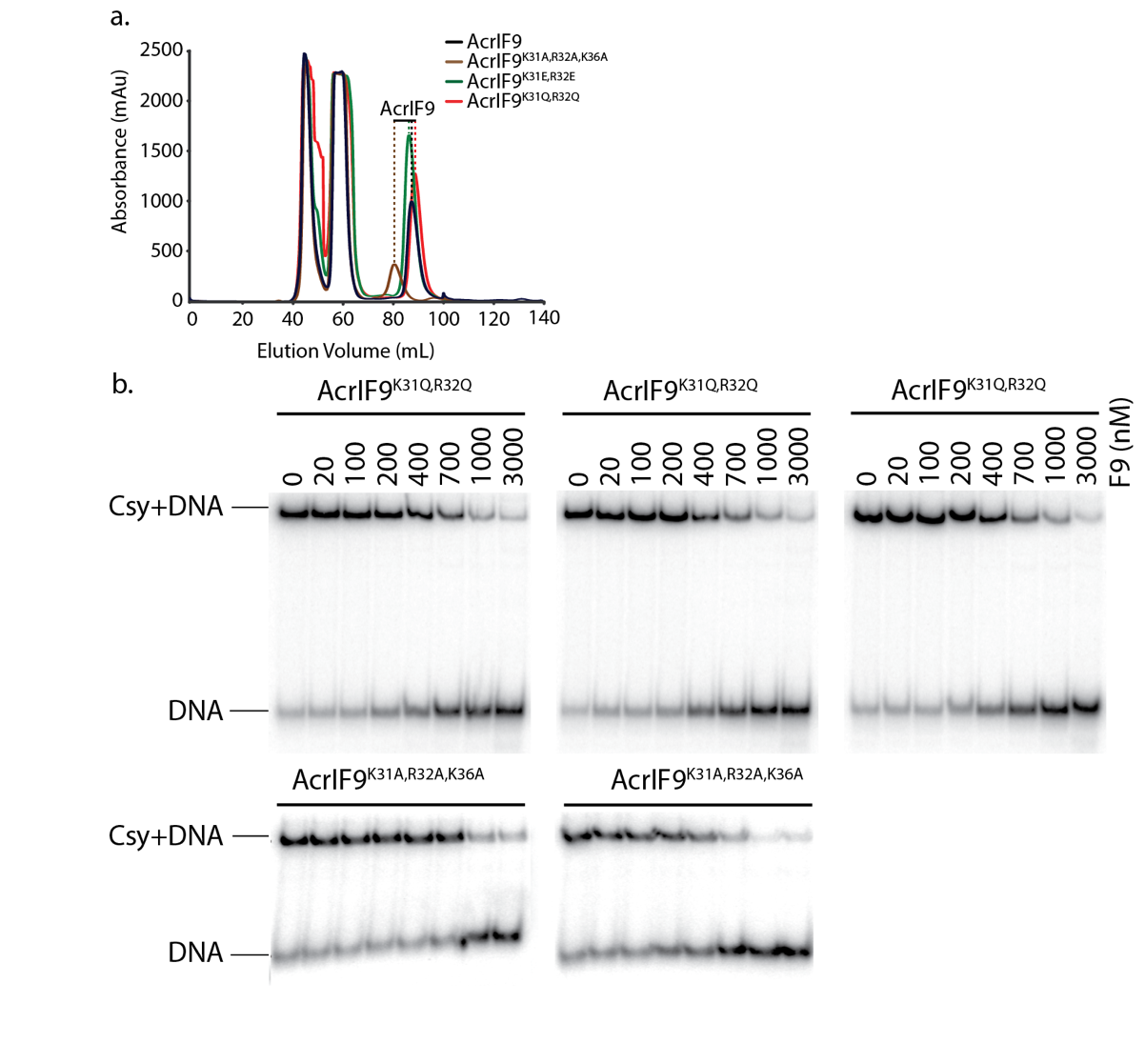
**

**Supplementary Fig. 6 AcrIF9 basic patch mutants have a folding defect.** **a.** Size exclusion chromatography profile of AcrIF9 mutants AcrIF9^K31A,R32A,K36A^, AcrIF9^K31Q,R32Q^ and AcrIF9^K31E,R32E^ showed altered size exclusion profiles. **b.** Electrophoretic mobility shift assays of AcrIF9 basic patch mutants do not inhibit target-DNA binding as AcrIF9 wildtype. Csy (200 nM) was incubated for 15 min with increasing concentrations of AcrIF9 prior to addition of ^32^P-labeled target DNA. Source data are provided as a Source Data file.

**Supplementary table 1: Cryo-EM data and refinement statistics.**

|  | Csy-AcrIF9  (EMDB-21516)  (PDB 6W1X) | Csy-AcrIF9-dsDNA  (EMDB-21517)  (PDB 6WHI) |
| --- | --- | --- |
| **Data collection and processing** |  |  |
| Microscope | Talos Arctica | Talos Arctica |
| Voltage (keV) | 200 | 200 |
| Nominal magnification | x36,000 | X36,000 |
| Exposure navigation | Image shift | Image shift |
| Electron exposure (e^-^ ^Å-2^) | 66 | 66 |
| Exposure rate (e^-^ pixel^-1^ s^-1^) | 5 | 5.5 |
| Detector | K2 Summit | K2 Summit |
| Defocus range (μm) | -0.8 to -1.2 | -0.8 to -1.2 |
| Pixel size (Å) | 1.15 | 1.15 |
| Micrographs | 1860 | 6472 |
| Automation software | Leginon | Leginon |
| Total extracted particles (no.) | 1,571,636 | 1,049,173 |
| Refined particles (no.) | 1,531,534 | 1,049,173 |
|  |  |  |
| **Reconstruction** |  |  |
| Final particles (no.) | 285,707 | 152,066 |
| Symmetry imposed | P1 | P1 |
| Map resolution (Å)  FSC 0.5 (unmasked/masked)  FSC 0.143 (unmasked/masked) | 3.9  4.7/4.3  4.0/3.9 | 4.2  4.8/4.6  4.4/4.2 |
| Map resolution range (Å) | 3.4-7.4 | 3.8-6.2 |
| Applied B-factor (Å^2^) | -103 | -150 |
|  |  |  |
| **Refinement** |  |  |
| Refinement package | Phenix | Phenix |
| Initial model used (PDB code) | 5UZ9 | 6W1X, 5UZ9 |
| Model composition  Protein residues  Nucleotide residues | 2875  60 | 2968  110 |
| Map Correlation Coefficient | 0.69 | 0.76 |
| *B* factors (Å^2^)  Protein | 65.5 | 61.6 |
| R.m.s. deviations  Bond lengths (Å)  Bond angles (°) | 0.09  1.189 | 0.006  1.161 |
| Ramachandran plot  Favored (%)  Allowed (%)  Disallowed (%) | 99.72  0.28  0 | 98.23  1.77  0 |
| Poor rotamers (%) | 0.06 | 0 |
| MolProbity score | 1.28 | 1.51 |
| Clashscore (all atoms) | 5.21 | 9.66 |
| C-beta deviations | 0 | 0 |
| CaBLAM outliers (%) | 2.00 | 2.49 |
| EMRinger score | 0.89 | 2.02 |
