# Supplementary material for: AcrIF9 tethers non-sequence specific dsDNA to the CRISPR RNA-guided surveillance complex
Source: Nat Commun. 2020 Jun 1;11:2730. doi: 10.1038/s41467-020-16512-1 (PMC7264359; doi:10.1038/s41467-020-16512-1)
Supplement: Supplementary file 3 — Reporting Summary [file 41467_2020_16512_MOESM3_ESM.pdf]

nature research

Corresponding author(s): Alan R. Davidson, Gabriel C. Lander, Blake Wiedenheft

Last updated by author(s): Apr 6, 2020

Reporting Summary

Nature Research wishes to improve the reproducibility of the work that we publish. This form provides structure for consistency and transparency in reporting. For further information on Nature Research policies, see Authors & Referees and the Editorial Policy Checklist .

Statistics

For all statistical analyses, confirm that the following items are present in the figure legend, table legend, main text, or Methods section.

n/a	Confirmed	
☐	☒	The exact sample size (n) for each experimental group/condition, given as a discrete number and unit of measurement
☒	☐	A statement on whether measurements were taken from distinct samples or whether the same sample was measured repeatedly
☒	☐	The statistical test(s) used AND whether they are one- or two-sided<br>*Only common tests should be described solely by name; describe more complex techniques in the Methods section.*
☒	☐	A description of all covariates tested
☒	☐	A description of any assumptions or corrections, such as tests of normality and adjustment for multiple comparisons
☒	☐	A full description of the statistical parameters including central tendency (e.g. means) or other basic estimates (e.g. regression coefficient) AND variation (e.g. standard deviation) or associated estimates of uncertainty (e.g. confidence intervals)
☒	☐	For null hypothesis testing, the test statistic (e.g. F, t, r) with confidence intervals, effect sizes, degrees of freedom and P value noted<br>*Give P values as exact values whenever suitable.*
☒	☐	For Bayesian analysis, information on the choice of priors and Markov chain Monte Carlo settings
☒	☐	For hierarchical and complex designs, identification of the appropriate level for tests and full reporting of outcomes
☒	☐	Estimates of effect sizes (e.g. Cohen's d, Pearson's r), indicating how they were calculated

Our web collection on statistics for biologists contains articles on many of the points above.

Software and code

Policy information about availability of computer code

Data collection	Cryo-EM data were collected and processed using the Leginon and Appion pipelines as described in the methods.
Data analysis	Frame alignment, CTF estimation and particle picking were performed with MotionCorr, Gctf and Dogpicker, respectively. 2D classification and generation of the initial 3D models were performed in CryoSparc. Relion was used for 3D auto-refinement, as described in the methods.

For manuscripts utilizing custom algorithms or software that are central to the research but not yet described in published literature, software must be made available to editors/reviewers. We strongly encourage code deposition in a community repository (e.g. GitHub). See the Nature Research guidelines for submitting code & software for further information.

Data

Policy information about availability of data

All manuscripts must include a data availability statement. This statement should provide the following information, where applicable:

- Accession codes, unique identifiers, or web links for publicly available datasets
- A list of figures that have associated raw data
- A description of any restrictions on data availability

The cryo-EM maps of Csy-AcrIF9 and Csy-AcrIF9-dsDNA have been deposited in the Electron Microscopy Data Bank (EMDB) under the accession codes EMD-21516 and EMD-21517, respectively. Atomic coordinates of the models have been deposited in the Protein Data Bank (PDB) under accession codes 6W1X [https://www.rcsb.org/structure/6W1X] and 6WHI [https://www.rcsb.org/structure/6WHI].

Field-specific reporting

Please select the one below that is the best fit for your research. If you are not sure, read the appropriate sections before making your selection.

☒ Life sciences ☐ Behavioural & social sciences ☐ Ecological, evolutionary & environmental sciences

For a reference copy of the document with all sections, see nature.com/documents/nr-reporting-summary-flat.pdf

Life sciences study design

All studies must disclose on these points even when the disclosure is negative.

Sample size	Gels in figure 2 were repeated in triplicate but the data is not quantified and statistical analysis has not been preformed. These gels are only intended to demonstrate a binding behavior. An accurate quantitative evaluation will required considerably more work due to the complex nature of several simultaneous interactions.
Data exclusions	Results in the gel shift assays are buffer dependent. Gels and results generated during buffer optimization have been excluded.
Replication	Gels were repeated in triplicate
Randomization	There were no experimental subjects in this study.
Blinding	Biochemistry was performed in one lab and the structures were determined in another. Structure interpretation was a joint effort, but none of the authors were blinded for any part of this work.

Reporting for specific materials, systems and methods

We require information from authors about some types of materials, experimental systems and methods used in many studies. Here, indicate whether each material, system or method listed is relevant to your study. If you are not sure if a list item applies to your research, read the appropriate section before selecting a response.

Materials & experimental systems

n/a	Involved in the study	
☒	☐	Antibodies
☒	☐	Eukaryotic cell lines
☒	☐	Palaeontology
☒	☐	Animals and other organisms
☒	☐	Human research participants
☒	☐	Clinical data

Methods

n/a	Involved in the study	
☒	☐	ChIP-seq
☒	☐	Flow cytometry
☒	☐	MRI-based neuroimaging
